# Characterization of porcine endogenous retrovirus insertion in Jeju native pigs and commercial breeds

**DOI:** 10.5713/ab.25.0174

**Published:** 2025-06-10

**Authors:** Seungwon Yoon, Mrinmoy Ghosh, Myeongyeon Shin, Hyunyong Choi, Cheol-Ho Hyun, Dae Cheol Kim, Shin Ji Lee, Min Jee An, Young-Ok Son, Chang-Gi Hur

**Affiliations:** 1 Interdisciplinary Graduate Program in Advanced Convergence Technology and Science, Jeju National University, Jeju, Korea; 2 Saint One Ltd., Jeju, Korea; 3 Department of Animal Biotechnology, Faculty of Biotechnology, College of Applied Life Sciences, Jeju National University, Jeju, Korea; 4 Livestock and Life Sciences Research Institute, Jeju, Korea; 5 Department of Physical Education, Korea National University of Education, Cheongju, Korea; 6 Bio-Health Materials Core-Facility Center, Jeju National University, Jeju, Korea; 7 Practical Translational Research Center, Jeju National University, Jeju, Korea

**Keywords:** Jeju Native Pig (JNP), Porcine Endogenous Retrovirus (PERV), Whole-genome Sequencing, Xenotransplantation

## Abstract

**Objective:**

This study aimed to characterize the genomic distribution and amino acid homology of porcine endogenous retrovirus (PERV) subtypes in three pig breeds, Jeju native pigs (JNPs), Duroc, and Landrace.

**Methods:**

Genomic DNA was extracted from hair and ear tissue samples of JNPs, Duroc, and Landrace breeds using DirEx Fast Hair Kit and Exgene Tissue SV Plus kit (GeneAll). Whole-genome resequencing (WGR) was performed by using the Illumina NovaSeq 6000 platform. Sequencing libraries were prepared using the TruSeq Nano DNA Kit and quality-checked using QUAST and BUSCO, and aligned to the *Sus scrofa* 11.1 reference genome with Bowtie2. Polymerase chain reaction (PCR) and quantitative real-time PCR were conducted with subtype-specific primers targeting *gag*, *pol*, and *env* regions. Amplicons were verified via agarose gel electrophoresis, purified, and subjected to Sanger sequencing.

**Results:**

WGR revealed breed-specific differences in PERV insertion, with JNPs exhibiting a higher frequency compared with the commercial breeds. PERV-B was the most abundant subtype, followed by PERV-CA and PERV-A, whereas PERV-C was absent in all the breeds. Chromosomal mapping highlighted variations in the localization of PERV, with notable absence on chromosomes 10 and 18. Homology analysis of amino acid sequences of PERV-A, PERV-B, and PERV-CA revealed breed-specific variations in the *gag*, *pol*, and *env* regions, indicating potential differences in viral replication and infectivity. The presence of all PERV subtypes were confirmed using polymerase chain reaction, with PERV-C detected in some Western breeds and all the JNPs analyzed. Sequencing of the PERV-C *env* region revealed single nucleotide polymorphisms, indicating genetic divergence among pig breeds.

**Conclusion:**

The study findings highlight the need for breed-specific strategies in PERV inactivation for xenotransplantation applications. The distinct chromosomal distribution patterns and functionally significant PERV insertions identified in this study provide a foundation for future research into host–virus interactions and retroviral evolution.

## INTRODUCTION

Advancements in genetically engineered pigs have ushered in new possibilities for xenotransplantation. However, potential transmission of animal-derived pathogens remains a significant challenge in transplantation [1]. Xenotransplantation can enable pathogenic microorganisms to cause unexpected infections in new hosts, posing health risks to recipients and the society as a whole. Therefore, monitoring and managing animal pathogens is crucial for the safe and effective implementation of xenotransplantation [2]. Direct contact between body secretions and blood is the primary route of infection in pigs. Common zoonotic pathogens include Salmonella, *Escherichia coli*, Streptococcus, the hepatitis E virus, the Japanese encephalitis virus, and the Nipah virus. Strict biosecurity measures, such as vaccination, antiviral administration, and rearing in specific pathogen-free facilities, have been implemented to prevent the transmission of exogenous pathogens [2]. However, unlike exogenous pathogens, endogenous retroviruses (ERVs) are integrated into the host genome and inherited across generations because of which their elimination through external management strategies is impossible [3].

Porcine endogenous retroviruses (PERVs) are classified within the Retroviridae family, Orthoretrovirinae subfamily, Gammaretrovirus genus, and Porcine type-C oncovirus species. These viruses are estimated to have been existing for approximately 7–8 million years [4]. The PERV genome consists of a single-stranded RNA, with both coding and noncoding sequences. The coding region includes three essential genes: *gag*, which encodes viral structural proteins; *pol*, which encodes enzymes required for viral replication; and *env*, which encodes envelope proteins responsible for receptor binding and infection of host cells [5]. The *env* gene plays a critical role in PERV infectivity and is used for subtype classification [6]. Among the identified PERV subtypes, only PERV-A, PERV-B, and PERV-C are replication-competent. PERV-A and PERV-B can infect both pig and human cells, whereas PERV-C is restricted to pig cells [7]. Additionally, recombinant PERV-A/C (PERV-CA) increases the infectivity in human cells, raising concerns about potential cross-species transmission during xenotransplantation [8].

Substantial progress has been made in xenotransplantation research. In September 2021, Cooper [9] transplanted a gene-edited pig kidney into a brain-dead human, achieving normal renal function for 54 h without hyperacute rejection. Later, Porrett et al [10] implanted a kidney from a pig with 10 gene edits, including deletion of GGTA1, CMAH, and β4GALNT2. The donor pig carried PERV-A and PERV-B but lacked PERV-C. The graft remained structurally intact and free of hyperacute rejection for 74 h; however, the recipient succumbed to systemic organ failure [9,10]. In January 2022, Griffith et al [11] transplanted a genetically edited pig heart into a patient with end-stage heart failure; the graft worked for seven weeks, but porcine cytomegalovirus detected on day 43 contributed to the patient’s death on day 60. In March 2023, surgeons from Massachusetts General Hospital implanted a kidney from a PERV-inactivated, gene-edited pig [12]; though the renal function remained stable, the recipient died of unrelated causes approximately 60 days post transplant. Although no human xenotransplant recipient has shown direct PERV infection, brief survival times limit long-term surveillance. The clarification on PERV safety thus remains uncertain. Comprehensive inactivation of all PERV loci in donor pigs via multiplex gene editing is therefore viewed as a pivotal step toward safer, more feasible xenotransplantation.

Among the various animal models, Jeju native pigs (JNPs) represent a valuable genetic resource owing to their unique traits and potential biomedical applications. However, similar to other pig breeds, JNPs harbor ERVs that are integrated into their genome and inherited via germline transmission. Unlike exogenous pathogens, which can be controlled through strict biosecurity measures, ERVs cannot be completely eliminated through external interventions [3]. This poses a challenge for xenotransplantation because PERVs can potentially infect human cells, leading to concerns regarding cross-species transmission.

This study was aimed at analyzing the presence, distribution, and genetic characteristics of PERVs in JNPs. By examining the genomic composition and expression of PERVs in JNPs, we aimed to provide insights into their potential impact on xenotransplantation and to contribute to the development of safer and more effective transplantation practices.

## MATERIALS AND METHODS

### Whole-genome sequencing

This study’s protocol was reviewed and approved by the Institutional Animal Care and Use Committee (IACUC) of Jeju National University (No. 2024-0079). Genomic DNA was extracted from hair samples collected from three JNPs (J-17, J-23, and J-24), three Duroc pigs (D-41, D-42, and D-45), and three Landrace pigs (L-9, L-21, and L-34) using the DirEx Fast Hair Kit (GeneAll), according to the manufacturer’s instructions. The hair sample was placed in a DirEx Fast-Hair tube and was subjected to thermal processing in a thermal cycler; it was incubated at 65°C for 5 min and subsequently at 95°C for 5 min to enhance DNA release. After extraction, the supernatant (excluding hair remnants) was transferred to a sterile centrifuge tube and DNA purity and concentration were assessed using a NanoDrop spectrophotometer (Thermo Fisher Scientific). DNA integrity was confirmed via electrophoresis on a 1.5% agarose gel. The final DNA concentration ranged from 20 to 50 ng/μL, with an average purity ratio (A260/A280) of 1.8–2.0, indicating high-quality DNA suitable for sequencing.

Whole-genome resequencing (WGR) was performed at Macrogen using a next-generation sequencing (NGS) technology. A sequencing library was constructed using the TruSeq Nano DNA Kit (Illumina), targeting a 350 bp insert size. Paired-end sequencing (2×150 bp) was performed using the Illumina NovaSeq 6000 platform, generating an average raw sequencing depth of 30× per sample. The total sequencing output across the nine samples yielded approximately 900 Gb of raw data, ensuring comprehensive genome coverage for subsequent analysis.

### Analysis of porcine endogenous retrovirus integration in genetically distinct pig breeds

The raw NGS data for each specimen were processed using DNASTAR Lasergene v17.0 for quality control and downstream analysis. Initial data quality assessment was conducted using the Quality Assessment Tool for Genome Assemblies (QUAST v5.2.0) and Benchmarking Universal Single-Copy Orthologs (BUSCO v5.4.3), with completeness scores exceeding 95% across samples, ensuring high-quality sequencing reads. The reference genome, *Sus scrofa* 11.1 (GCF_ 000003025.6), was obtained from the NCBI database. Raw sequencing reads were aligned to the reference genome using Bowtie2 v2.4.5, achieving an average mapping rate of 98.6% per sample. The resulting SAM files were converted to the BAM format using SAMtools v1.15, and the genome coordinates were indexed to identify specific genomic regions of interest. For variant annotation, ANNOVAR and Genome Analysis Toolkit (GATK v4.2.6.1) were used with stringent filtering criteria, such as phred quality score (≥30), read depth (≥10), and minor allele frequency (≤0.05).

The reference sequences for PERV subtypes A (AY099323), B (AY099324), C (KC116221), and recombinant A/C (AY953542) were used for subtype identification. To refine the subtype classification, approximately 3 kb of flanking sequences around the candidate PERV loci were retrieved and aligned to the respective PERV reference sequences. The homology-rich *gag*-*pol* region (>90% sequence similarity) was aligned to each individual genome using SnapGene v6.1, enabling detection of the number and genomic positions of PERV insertions. To refine the subtype classification, approximately 3 kb of flanking sequences surrounding each candidate PERV locus were retrieved and aligned against the respective PERV reference sequences. Subtype identification and full-length sequence verification were conducted by comparing homology within the env-3′ long terminal repeat region, ensuring subtype accuracy. Finally, amino acid sequences corresponding to the *gag*, *pol*, and *env* regions of the identified PERVs were translated and aligned using benchling. Sequence homology within key functional domains was analyzed to assess potential differences in infectivity among the JNP, Duroc, and Landrace breeds.

### Quantitative real-time polymerase chain reaction analysis and sequencing

Ear tissue samples (20–30 mg) were collected from three JNPs, three Landrace pigs, and three Duroc pigs under sterile conditions. Genomic DNA was extracted using the Exgene Tissue SV Plus kit (GeneAll) following the manufacturer’s protocol. DNA concentration and purity were assessed using a NanoDrop 2000 spectrophotometer (Thermo Fisher Scientific), and only samples with an A260/A280 ratio between 1.8 and 2.0 were selected for further analysis to ensure that the high-quality DNA was suitable for polymerase chain reaction (PCR) amplification. PCR amplification was performed using Han Taq DNA Polymerase (HanLAB) with PERV subtype-specific primers targeting the *gag, pol*, and *env* regions. Primer sequences were designed based on previously reported conserved regions for PERV subtypes A (AY099323), B (AY099324), C (KC116221), and recombinant A/C (AY953542) (Table 1).

The amplicon sizes for PERV-A, -B, -C, and -A/C were approximately 650, 720, 550, and 800 bp, respectively. Each 25 μL PCR mixture contained 50 ng of genomic DNA, 0.2 μM each of forward and reverse primers, 200 μM dNTPs (Takara), 1× PCR buffer (Mg^2+^ included, HanLAB), and 0.25 U Han Taq DNA polymerase. Both conventional PCR and quantitative real-time polymerase chain reaction (qRT-PCR) were performed using an iCycler Thermal Cycler (Agilent Technologies) at the Bio-Health Materials Core Facility of Jeju National University. The thermocycling conditions were as follows: initial denaturation at 95ºC for 5 min, followed by 36 cycles of amplification consisting of denaturation at 95°C for 30 s, annealing at 60°C for 30 s, extension at 72°C for 1 min, and a final extension at 72°C for 5 min. For qRT-PCR, SYBR Green-based detection (Takara Bio) was used with fluorescence acquisition at each cycle, and melt curve analysis was performed to confirm amplicon specificity. The PCR products were resolved via electrophoresis on a 2% agarose gel in 1× TAE buffer at 100 V for 45 min and visualized under UV light using a GelDoc^XR+^ imaging system (Bio-Rad) after staining the gel with GelRed (Biotium). Amplicons of the expected size were excised, purified using the Expin PCR SV kit (GeneAll), and subjected to Sanger sequencing at Macrogen, Inc. Sequencing was performed in both the forward and reverse directions with the same primer sets that were used for PCR amplification. The obtained sequences were analyzed using SnapGene (Insightful Science) for alignment with the reference sequences, and sequence homology was confirmed using BLAST (NCBI). Phylogenetic analysis was conducted using MEGA X (Molecular Evolutionary Genetics Analysis software) with the neighbor-joining method, and bootstrap resampling was performed with 1,000 replicates to assess phylogenetic confidence.

## RESULTS

### Distribution of porcine endogenous retrovirus subtypes in genetically distinct pig breed chromosomes

**Figure 1:**
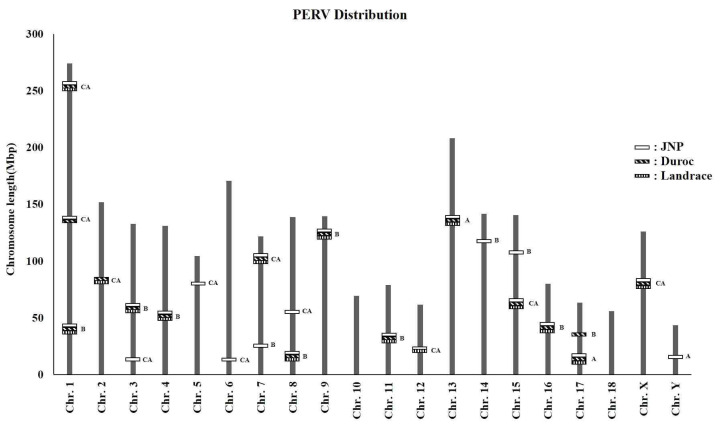
Chromosomal mapping of PERV insertions in JNP, Duroc, and Landrace. Chromosomal mapping shows the genomic locations of PERVs in the JNP, Duroc, and Landrace breeds. Chromosomal scaffolds are indicated in gray, with empty rectangles, rectangles with diagonal lines, and rectangles with vertical lines showing PERV insertions in JNP, Duroc, and Landrace chromosomes. For simplicity, the orientation of the genes was omitted, and both full-length and truncated forms (short and long PERV genes) were collectively mapped. This mapping highlights the distribution of PERV types, which may affect xenotransplantation outcomes. A: PERV-A type; B: PERV-B type; CA: PERV-CA type. PERV, porcine endogenous retrovirus; JNP, Jeju native pig.

Systematic analysis of the chromosomal distribution of PERV subtypes in the JNP, Duroc, and Landrace breeds revealed breed-specific differences in PERV insertion across the genomes (Figure 1; Table 2), highlighting significant disparities in the frequency and chromosomal localization of PERV insertion, contributing to the understanding of retroviral integration patterns in different pig lineages. Notably, JNPs exhibited a significantly higher frequency of PERV insertion than the two Western commercial breeds, Duroc and Landrace. Among the PERV subtypes identified, PERV-B was the most abundant, followed by PERV-CA, and PERV-A. However, PERV-C was absent in all three breeds, suggesting either a historical loss of this subtype or a selective disadvantage that led to its elimination. This pattern suggests that JNPs have undergone unique evolutionary pressures that favor the retention of certain PERV insertions, potentially influencing their genomic stability and resistance to viral activation. Moreover, PERV insertion was not observed on chromosomes 10 and 18 across all breeds despite its presence on multiple chromosomes.

### Comparative analysis of amino acid homology in the *gag, pol*, and *env* regions of porcine endogenous retrovirus-A

Comparative evaluation of amino acid sequences in the PERV-A gag, pol, and env regions revealed breed-specific variations, indicating potential functional differences among the examined pig breeds. This analysis highlighted critical differences in the distribution, homology, and potential replication capacity of PERV-A elements among the JNP, Duroc, and Landrace breeds. JNPs exhibited three instances of PERV-A insertion, whereas the Duroc and Landrace breeds contained two insertions each (Table 3).

**Figure 2:**
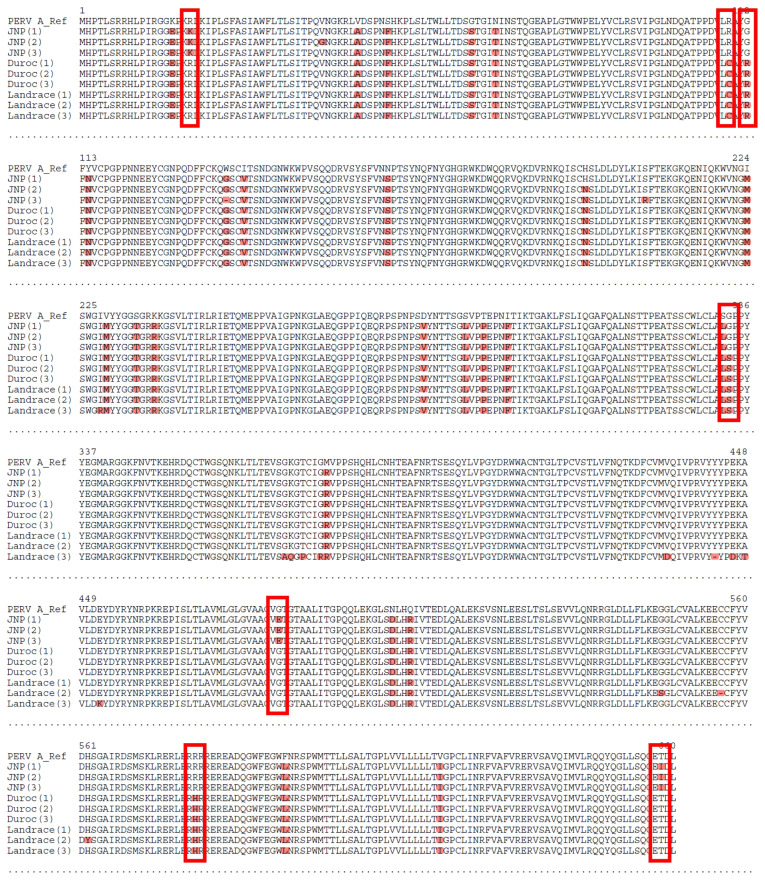
Alignment of deduced amino acid sequences of PERV-A env. The deduced amino acid sequences of PERV-A env were aligned. The sequences from the JNP, Duroc, and Landrace env strains were compared with those of the reference strain (PERV-A: ay099323). Seven amino acid differences were identified in PERV-A between the sequences from JNP and the two Western pig breeds, indicating potential variations in xenoreactivity affecting the immune compatibility of xenografts. PERV, porcine endogenous retrovirus; JNP, Jeju native pig.

The chromosomal locations of PERV-A insertion vary among breeds, with significant implications for viral function and host–virus interactions. Only PERV-A insertion in chromosomes 13 (JNPs) and 17 (Duroc and Landrace) demonstrated high sequence homology with the pol region of the reference sequence. The pol region encodes critical viral enzymes, including reverse transcriptase, integrase, and RNase H, which are essential for proviral DNA synthesis and integration into the host chromosomes. The env region, which encodes viral envelope glycoproteins that mediate host cell entry, also exhibited substantial sequence homology across all breeds. However, seven amino acid substitutions were observed in the JNPs compared with those in the Duroc and Landrace breeds (Figure 2). As these variations may affect receptor binding affinity, host cell tropism, or immune evasion, further experimental validation is warranted to determine their functional consequences. The high homology observed in the *pol, env*, and *gag* regions of PERV-A exhibited low sequence homology across all breeds compared with the reference genome. The PERV-A insertion was identified exclusively on the Y chromosome in JNPs, a feature absent in the two Western breeds. However, the Y-linked PERV-A exhibited reduced homology in its *pol* region, indicative of a potential loss of replicative capacity.

### Comparative analysis of amino acid homology in the *gag, pol*, and *env* regions of porcine endogenous retrovirus-B across pig breeds

Analyses of chromosomal distribution of PERV-B insertion and sequence homology revealed breed-specific variations, indicating potential differences in viral replication, infectivity, and evolutionary adaptation. PERV-B insertion was identified in multiple chromosomes across the examined breeds, with JNPs harboring insertions on chromosomes 3, 4, and 16; Duroc pigs on chromosomes 3, 4, 9, and 11, and Landrace pigs on chromosomes 3, 4, and 9 (Table 4). All these insertions retained high sequence homology with the pol region of the reference genome, implying that these elements still possess the self-replicative potential. PERV-B insertion in chromosome number 4 showed high homology in the gag and pol regions across all breeds, but the *env* region in JNPs exhibited significant divergence compared with Western breeds. PERV-B insertion in chromosomes number 9 and 11 exhibited high *pol* homologies in the Duroc and Landrace breeds, suggestive of retained replicative potential, whereas JNPs showed relatively reduced homology in these regions. This interbreed variation in PERV-B genomic characteristics indicates that JNPs may possess unique viral adaptations that differentiate them from the Western breeds.

### Comparative analysis of amino acid homology in the *gag, pol*, and *env* regions of porcine endogenous retrovirus-CA

Analyses of chromosomal distribution of PERV-CA insertion and sequence homology revealed distinct patterns for the examined pig breeds, indicating variations in the viral replication potential and infectivity. PERV-CA insertion was observed in multiple chromosomes, with JNPs harboring insertions on chromosomes 3 and 5, Duroc pigs on chromosomes 1 and 12, and Landrace pigs on chromosome 1 (Table 5).

**Figure 3:**
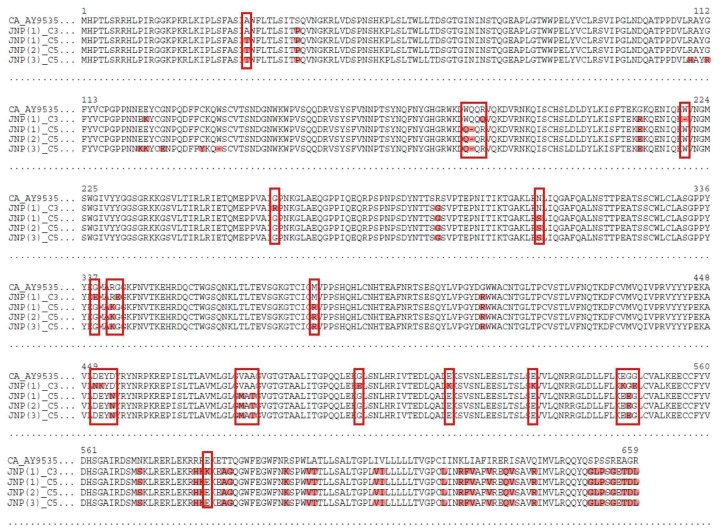
Alignment of deduced amino acid sequences of PERV-CA env. The deduced amino acid sequences of PERV-CA env were aligned. The PERV-CA sequences from JNP chromosomes 3 and 5 were compared with those of the reference strain (PERV-CA: ay953542). Twenty-three amino acid differences were identified in PERV-CA between the sequences from JNP chromosomes 3 and 5, suggesting a functional divergence that could affect viral infectivity and immune evasion in the context of xenotransplantation. JNP, Jeju native pig; PERV, porcine endogenous retrovirus.

PERV-CA insertion in chromosomes 3 and 5 of JNPs exhibited high homology in the gag, pol, and env regions compared with the reference sequence, indicating that these elements may have retained their functional integrity. However, multiple amino acid differences were identified within the *env* region (Figure 3), indicating potential variations in viral receptor-binding affinity and host cell tropism. PERV-CA insertion in chromosome 1 of Duroc and Landrace pigs demonstrated high homology with the reference *pol* and *env* regions but contained significant deletions in the *gag* region. The gag gene encodes structural proteins essential for viral assembly and capsid formation. Therefore, such deletions could result in incomplete or defective viral particles, reducing their ability to form fully functional virions. PERV-CA insertion was observed on chromosome 12 in both JNP and Duroc pigs, and only Duroc-derived PERV-CA retained high homology with the reference pol sequence. This suggests that the Duroc strain may still harbor an active form of PERV-CA, whereas the JNP variant may have accumulated mutations, leading to reduced replication efficiency.

### Detection of porcine endogenous retrovirus subtypes and sequence alignment of the *env* region of porcine endogenous retrovirus C in Jeju native pig

**Figure 4:**
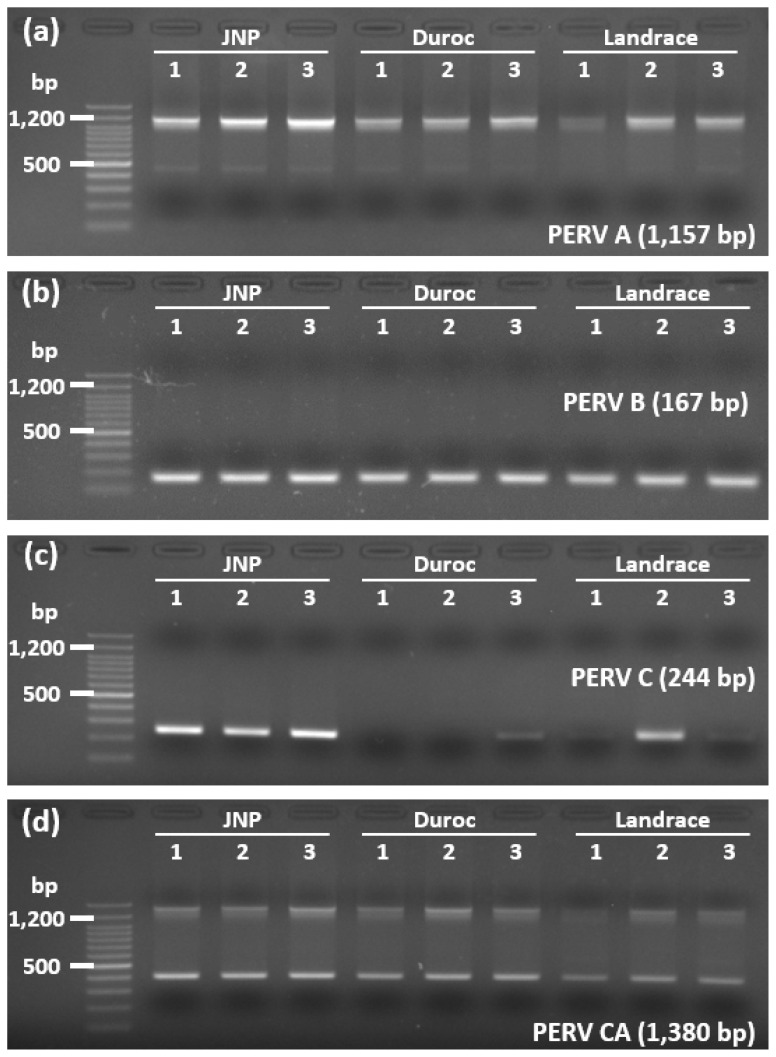
Detection of PERV subtypes (A, B, C, and CA) via PCR in Jeju native pigs and Duroc and Landrace breeds. The PCR products were obtained using subtype-specific primers targeting PERV A (1,157 bp), PERV B (167 bp), PERV C (244 bp), and PERV CA (1,380 bp). Amplicons were visualized on 2% agarose gels, with a DNA size marker on the left. The amplicons for PERV A, B, and CA were consistent with the whole-genome resequencing results, whereas those for PERV C showed variations depending on the breed. JNP, Jeju native pig; PERV, porcine endogenous retrovirus; PCR, polymerase chain reaction.

All PERV subtypes (PERV-A, PERV-B, PERV-C, and PERV-CA) were successfully detected using PCR analysis across the examined pig breeds, confirming their genomic presence (Figure 4). PCR amplification was performed using subtype-specific primers, which generated distinct amplicons corresponding to PERV-A (1,157 bp), PERV-B (167 bp), PERV-C (244 bp), and PERV-CA (1,380 bp). The PCR products were visualized via electrophoresis on a 2% agarose gel, revealing consistent results for PERV-A, PERV-B, and PERV-CA, in concordance with the WGR data. However, the presence of PERV-C varies among breeds, suggesting potential breed-specific differences in its genomic distribution and expression.

**Figure 5:**
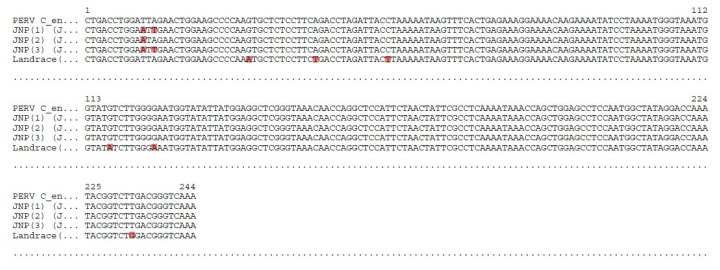
Sequence alignment of the PERV C env region amplified from Jeju native pigs and Landrace breeds, where PERV C was detected. The alignment compared nucleotide sequences from three Jeju native and one Landrace pigs with the reference PERV C env sequence (244 bp). Variations are highlighted in red, indicating single nucleotide polymorphisms within the analyzed region. The alignment confirmed successful amplification and sequence specificity of the PERV C env region. JNP, Jeju native pig; PERV, porcine endogenous retrovirus.

Sequencing analysis of the amplified *env* region was performed to confirm the presence of PERV-C. Sequence alignment of the *env* region from JNPs and a Landrace pig verified the specificity of amplification (Figure 5). Comparison of the 244 bp nucleotide sequences from three JNPs and one Landrace pig with the reference PERV-C *env* sequence revealed the presence of nucleotide polymorphisms.

## DISCUSSION

PERV infection in human cell lines prompted significant efforts to characterize their genetic structure and suppress infectivity [13,14]. Subsequent *in vivo* investigations in immunodeficient mice revealed the ability of PERVs to integrate into the host genome, which led to attempts at mitigating the risks associated with the use of antiretroviral drugs, neutralizing antibodies, and RNA interference [15–18]. Moreover, the discovery of PERV recombination events has raised concerns regarding their evolutionary adaptability and implications for human infections [19]. In Korea, foundational studies on the distribution of PERVs in domestic pigs was initiated in 2004 with the analysis of the *pol* gene sequence. Subsequent studies have focused on cloning and characterizing PERV-A and PERV-B *env* genes from gnotobiotic pigs [20], molecular characterization of the *gag* gene of ERVs in domestic pigs [21], and phylogenetic analysis of ERV envelope genes in Korean pig populations [22]. These studies provided insights into the molecular and phylogenetic characteristics of PERVs, which are critical for enhancing the safety of porcine tissues in xenotransplantation models. Furthermore, the regulation of recombinant PERV-A/C infectivity via the C-terminal outer membrane glycoprotein of the PERV-C subtype [23] has been explored and genomic insertion sites between Asian and European pigs have been compared [24]. PERV subtypes have been classified using PCR [25] and RNA interference has been employed to suppress PERV expression, further increasing the safety of pig tissues for xenotransplantation. However, research on JNPs remains limited, as most studies have focused on foreign breeds, such as Large White, Landrace, and Duroc, raised in Korea [26,27].

Unlike conventional genes, PERVs exhibit breed-specific variations in their insertion sites, copy number, and chromosomal integration patterns. Differences in PERV insertion sites have been reported among European, Asian, and wild pig populations [28,29]. In this study, we employed WGR to analyze PERV insertion sites and copy number in JNPs, Duroc, and Landrace breeds. We identified 24 PERVs in JNPs, 18 in Duroc pigs, and 15 in Landrace pigs. The distribution of PERV subtypes indicated that PERV-B and PERV CA were prevalent across the three breeds, whereas PERV-A was present at a low frequency, and PERV C was undetectable. Because PERV-C does not infect human-derived cell lines, pigs harboring PERV-C may offer advantages as xenotransplantation models. However, the low frequency of PERV-C and potential recombination risks with other PERV subtypes necessitate additional inactivation strategies for safe xenotransplantation.

Comparative analysis of amino acid sequences inferred from WGR revealed significant variations in the PERV subtypes across pig breeds. The majority of PERVs display diverse mutations in the *pol* region, indicating that only a subset of PERVs remains functionally active. Moreover, differences were observed in amino acid sequences for the *gag* and *env* regions, even among PERVs predicted to be active, indicating potential breed- and individual-specific differences in viral structure and infectivity.

The increased prevalence of PERV insertions in JNPs may be attributable to their distinct evolutionary history, characterized by prolonged isolation and reduced selective breeding compared with that of commercial breeds. The genomic retention of PERV elements suggests possible adaptive advantages, such as immune modulation or resistance to viral infections, which warrant further investigation. Given that PERV-C is associated with recombination risks and increased zoonotic transmission potential, its absence in JNPs, Duroc, and Landrace pigs may be advantageous for xenotransplantation. This aligns with the findings of previous studies, indicating that PERV-C-containing genomes are more prone to generating replication-competent recombinant viruses, reinforcing the selection of genetically safer pig breeds for biomedical applications. WGR is a powerful tool for identifying genetic variations among individuals. However, it is subject to limitations, such as improper mapping and insufficient coverage of repetitive sequences or structurally complex regions. To address these challenges, we performed PCR validation for each PERV subtype. Our PCR results for PERV-A, B, and CA were consistent with WGR findings, while PERV-C was detected in some Western breeds and all three JNPs analyzed. The sequencing of amplified PERV-C PCR products confirmed alignment with the *env* region of PERV C. Previous studies have demonstrated discrepancies between NGS- and PCR-based detection due to sequencing biases, complex mutations, or low coverage in certain genomic regions [30].

The gag gene encodes essential structural proteins, including the matrix, capsid, and nucleocapsid proteins, which play critical roles in virion assembly and genome packaging. The pol region encodes enzymes, such as reverse transcriptase, integrase, and RNase H, which are vital for viral replication and host genome integration. In this study, PERV-B insertion in chromosome 3 exhibited high homology across the *gag, pol*, and *env* regions, suggestive of a conserved and potentially functional form of PERV-B that may contribute to viral persistence. However, whereas PERV-B insertion in chromosome 4 displayed conserved gag and pol regions across breeds, the *env* region in JNPs exhibited substantial divergence compared with the Western breeds. Given the role of *env* in viral entry, these sequence variations may influence receptor-binding affinity, host tropism, and infectivity. This suggests that PERV-B elements in JNPs undergo distinct evolutionary modifications that potentially affect their interactions with host cells. Further analysis of PERV-B insertions in chromosome 16 revealed high pol homology in JNPs, whereas Duroc and Landrace pigs exhibited reduced homology, implying a possible functional loss in the Western breeds. This suggests that JNPs may retain a more active replication capacity, reinforcing the notion that interbreed differences play a crucial role in the evolution of PERV and host–virus interactions. PERV-CA insertions in chromosomes 3 and 5 in JNPs exhibited high homology across *gag, pol*, and *env* regions, indicating functional integrity. However, variations in chromosomal locations imply potential differences in infectivity. The breed-specific differences in PERV-CA distribution underscore the complexity of retroviral evolution and their implications for cross-species transmission risks. The SNPs indicated potential sequence variations among individuals, suggesting genetic divergence in PERV-C among pig breeds. Validation of PERV subtypes via PCR and sequencing provides crucial evidence supporting the genomic characterization of PERV elements in different pig breeds. The detection of SNPs within the PERV-C *env* region highlights possible functional implications, warranting further investigation of their impact on viral infectivity, host specificity, and potential cross-species transmission.

## CONCLUSION

These findings underscore the need for breed-specific strategies for targeting PERVs for xenotransplantation applications. The distinct chromosomal distribution patterns observed in the three breeds suggest that genome editing approaches should be tailored to specific pig lineages to ensure effective PERV inactivation. Furthermore, the identification of functionally significant PERV insertions in certain genomic regions provides a valuable foundation for future research into their potential roles in host–virus interactions and retroviral evolution. By leveraging gene editing technologies, it may be possible to develop PERV-free JNPs, thereby enhancing their suitability as xenotransplantation models. Further research is crucial for improving the biosafety profile of pigs intended for biomedical applications and for mitigating the risks associated with PERV-mediated zoonotic transmission.

## Figures and Tables

**Table 1:** List of primers and Tm values

	Sequence (5′–3′)	Length (bp)	GC (%)	Tm (°C)	PCR product length (bp)
PERV_A_F	AAGAACAGAGGCCATCTCCTAAC	23	47.83	59.8	1,157
PERV_A_R	TTCTCCTTGGCTCAGAAGGC	20	55.00	59.7	
PERV_B_F	GGGCAAGTACAAAGTGATGAAAC	23	43.48	58.5	167
PERV_B_R	CTGCTCCCCCGCCATATTTA	20	55.00	59.6	
PERV_C_F	CTGACCTGGATTAGAACTGGAAGC	24	50.00	60.9	244
PERV_C_R	TTTGACCCGTCAAGACCGTA	20	50.00	59.0	
PERV_CA_F	AAGAACAGAGGCCATCTCCTAAC	23	47.83	59.8	1,380
PERV_CA_R	TTTCCCCTTCCACAAAGTTTTGTAC	25	40.00	60.1	

PCR, polymerase chain reaction; PERV, porcine endogenous retrovirus.

**Table 2:** Detection of PERV subtypes in different pig breeds

Breed	PERV-A	PERV-B	PERV-C	PERV-CA	Total
JNP	3	11	0	10	24
Duroc	2	8	0	8	18
Landrace	2	7	0	6	15

PERV, porcine endogenous retrovirus; JNP, Jeju native pig.

**Table 3:** Homology among PERV-A amino acid sequences from JNP, Duroc, and Landrace

Breed	Chromosome	N	Gag (%)	Pol (%)	Env (%)
JNP	Chr.13	3	45.50	97.95	96.27
	Chr.17	3	34.04	49.12	43.57
	Chr.y	3	94.37	86.73	64.00
Duroc	Chr.13	1	69.68	38.75	95.91
	Chr.17	3	15.74	95.46	96.58
Landrace	Chr.13	1	69.55	36.58	95.91
	Chr.17	3	18.18	97.85	95.10

PERV, porcine endogenous retrovirus; JNP, Jeju native pig.

**Table 4:** Homology among PERV-B amino acid sequences from JNP, Duroc, and Landrace

Breed	Chromosome	N	Gag (%)	Pol (%)	Env (%)
JNP	Chr.1	2	100.00	6.04	99.73
	Chr.3	2	99.56	99.00	99.34
	Chr.4	3	99.59	99.70	36.25
	Chr.7	1	74.37	41.21	13.07
	Chr.8	1	99.83	58.56	99.73
	Chr.9	3	99.81	89.95	87.83
	Chr.11	2	49.60	88.04	41.22
	Chr.14	2	21.80	77.75	98.87
	Chr.15	1	99.33	87.41	98.84
	Chr.16	3	99.82	99.50	99.69
	Chr.x	1	-	-	99.04
Duroc	Chr.1	1	99.85	25.39	99.59
	Chr.3	2	99.81	99.18	99.32
	Chr.4	3	99.86	99.73	37.57
	Chr.8	3	99.03	61.00	99.77
	Chr.9	3	99.58	99.67	87.25
	Chr.11	1	64.11	90.79	99.03
	Chr.16	1	99.85	27.90	-
	Chr.17	1	41.00	75.00	52.83
Landrace	Chr.1	2	100.00	10.94	99.51
	Chr.3	2	99.83	97.56	99.30
	Chr.4	3	99.82	99.40	38.94
	Chr.8	2	99.47	61.04	99.79
	Chr.9	3	99.64	99.65	87.02
	Chr.11	2	58.14	88.36	99.02
	Chr.16	2	8.55	12.02	99.30

PERV, porcine endogenous retrovirus; JNP, Jeju native pig.

**Table 5:** Homology among PERV-CA amino acid sequences from JNP, Duroc, and Landrace

Breed	Chromosome	N	Gag (%)	Pol (%)	Env (%)
JNP	Chr.1	1	42.39	66.05	31.61
	Chr.1(2)	3	98.08	49.87	82.82
	Chr.3	1	97.73	96.74	93.62
	Chr.5	3	97.84	98.47	94.02
	Chr.6	1	57.12	10.24	22.44
	Chr.7	1	98.30	59.85	58.32
	Chr.8	1	96.79	27.82	94.79
	Chr.12	2	63.14	41.37	69.23
	Chr.15	1	14.24	60.05	93.63
	Chr.x	3	96.10	45.78	94.08
Duroc	Chr.1	1	98.06	69.32	95.80
	Chr.1(2)	1	19.90	98.66	95.23
	Chr.2	2	20.86	53.83	39.16
	Chr.7	2	72.92	84.82	95.80
	Chr.10	2	14.96	37.06	52.90
	Chr.12	2	97.66	98.73	95.30
	Chr.15	3	17.74	15.10	93.38
	Chr.x	3	96.57	87.93	93.91
Landrace	Chr.1(2)	2	21.18	98.45	94.03
	Chr.2	3	23.27	53.05	38.78
	Chr.7	3	72.35	84.53	95.43
	Chr.10	3	14.49	40.15	52.52
	Chr.15	3	17.96	16.75	93.76
	Chr.x	3	96.32	87.49	93.75

PERV, porcine endogenous retrovirus; JNP, Jeju native pig.

## References

[1] Michler RE (1996). Xenotransplantation: risks, clinical potential, and future prospects. Emerg Infect Dis.

[2] Lin CN, Okabayashi T, Tummaruk P, Ooi PT (2022). Editorial: zoonotic diseases among pigs. Front Vet Sci.

[3] Choi J, Kim H, Yoon JK (2015). Identification of porcine endogenous retrovirus (PERV) packaging sequence and development of PERV packaging viral vector system. J Microbiol.

[4] Denner J, Tönjes RR (2012). Infection barriers to successful xenotransplantation focusing on porcine endogenous retroviruses. Clin Microbiol Rev.

[5] Blusch JH, Seelmeir S, von der Helm K (2002). Molecular and enzymatic characterization of the porcine endogenous retrovirus protease. J Virol.

[6] Güell M, Niu D, Kan Y (2017). PERV inactivation is necessary to guarantee absence of pig-to-patient PERVs transmission in xenotransplantation. Xenotransplantation.

[7] Harrison I, Takeuchi Y, Bartosch B, Stoye JP (2004). Determinants of high titer in recombinant porcine endogenous retroviruses. J Virol.

[8] Breese SS (1970). Virus-like particles occurring in cultures of stable pig kidney cell lines. Arch Virol.

[9] Cooper DKC (2021). Genetically engineered pig kidney transplantation in a brain-dead human subject. Xenotransplantation.

[10] Porrett PM, Orandi BJ, Kumar V (2022). First clinical-grade porcine kidney xenotransplant using a human decedent model. Am J Transplant.

[11] Griffith BP, Goerlich CE, Singh AK (2022). Genetically modified porcine-to-human cardiac xenotransplantation. N Engl J Med.

[12] Mallapaty S, Kozlov M (2024). First pig kidney transplant in a person: what it means for the future. Nature.

[13] Kim JH, Choi E, Jung ES (2009). Characterization of clones of human cell line infected with porcine endogenous retrovirus (PERV) from porcine cell line, PK-15. Infect Chemother.

[14] Akiyoshi DE, Denaro M, Zhu H, Greenstein JL, Banerjee P, Fishman JA (1998). Identification of a full-length cDNA for an endogenous retrovirus of miniature swine. J Virol.

[15] Karlas A, Kurth R, Denner J (2004). Inhibition of porcine endogenous retroviruses by RNA interference: increasing the safety of xenotransplantation. Virology.

[16] Fiebig U, Stephan O, Kurth R, Denner J (2003). Neutralizing antibodies against conserved domains of p15E of porcine endogenous retroviruses: basis for a vaccine for xenotransplantation?. Virology.

[17] Powell SK, Gates ME, Langford G (2000). Antiretroviral agents inhibit infection of human cells by porcine endogenous retroviruses. Antimicrob Agents Chemother.

[18] van der Laan LJW, Lockey C, Griffeth BC (2000). Infection by porcine endogenous retrovirus after islet xenotransplantation in SCID mice. Nature.

[19] Denner J, Schuurman HJ (2021). High prevalence of recombinant porcine endogenous retroviruses (PERV-A/Cs) in minipigs: a review on origin and presence. Viruses.

[20] Lee D, Lee J, Kwon M, Park HY, Kim YB (2004). Molecular cloning of PERV-A and PERV-B envelope genes from miniature pigs. J Bacteriol Virol.

[21] Lee J, Lee D, Yoo JY (2006). Molecular characterization of porcine endogenous retrovirus gag genes from pigs in Korea. J Bacteriol Virol.

[22] Lee D, Lee J, Uhm SJ (2006). Molecular characterization of the porcine endogenous retrovirus subclass A and B envelope gene from pigs. Transplant Proc.

[23] Kim SRM, Park SM, Lee KJ (2010). The infectivity of recombinant porcine endogenous retrovirus (PERV-A/C) is modulated by membrane-proximal cytoplasmic domain of PERV-C envelope tail. Korean J Microbiol.

[24] Jung WY, Kim JE, Jung KC (2010). Comparison of PERV genomic locations between Asian and European pigs. Anim Genet.

[25] Lee DH, Lee JE, Kim HM, Kim GW, Park HY, Kim YB (2007). Detection and classification of porcine endogenous retroviruses by polymerase chain reaction. J Anim Sci Technol.

[26] Lee YS, Son S, Lee HK, Lee RH, Shin D (2022). Elucidating breed-specific variants of native pigs in Korea: insights into pig breeds’ genomic characteristics. Anim Cells Syst.

[27] Chen JQ, Zhang MP, Tong XK (2022). Scan of the endogenous retrovirus sequences across the swine genome and survey of their copy number variation and sequence diversity among various Chinese and western pig breeds. Zool Res.

[28] Jung KC, Yu SL, Kim TH (2007). Insertional variations of two porcine endogenous retroviruses (PERVs) in Korean native pigs and Asian wild boars. Asian-Australas J Anim Sci.

[29] Jung WY, Yu SL, Seo DW (2012). Characterization of insertional variation of porcine endogenous retroviruses in six different pig breeds. Asian-Australas J Anim Sci.

[30] Kosugi S, Momozawa Y, Liu X, Terao C, Kubo M, Kamatani Y (2019). Comprehensive evaluation of structural variation detection algorithms for whole genome sequencing. Genome Biol.

